# Therapeutic Lifestyle Intervention Targeting Enhanced Cardiometabolic Health and Function for Persons with Chronic Spinal Cord Injury in Caregiver/Care-Receiver Co-Treatment: A Study Protocol of a Multisite Randomized Controlled Trial

**DOI:** 10.3390/ijerph20196819

**Published:** 2023-09-25

**Authors:** Gregory E. Bigford, Luisa F. Betancourt, Susan Charlifue, Mark S. Nash

**Affiliations:** 1 Department of Neurological Surgery, University of Miami Miller School of Medicine, Miami, FL 33136, USA; lbetancourt@med.miami.edu (L.F.B.); mnash@med.miami.edu (M.S.N.); 2 Craig Hospital, Englewood, CO 80113, USA; scharlifue@craighospital.org; 3 Department of Physical Medicine & Rehabilitation, University of Miami Miller School of Medicine, Miami, FL 33101, USA; 4 Department of Physical Therapy, University of Miami Miller School of Medicine, Miami, FL 33136, USA

**Keywords:** SCI, cardiometabolic disease, intervention, rehabilitation, exercise, nutrition, metabolism, behavioral modification

## Abstract

Background: Chronic spinal cord injury (SCI) significantly accelerates morbidity and mortality, partly due to the increased risk of cardiometabolic diseases (CMD), including neurogenic obesity, dyslipidemia, and impaired glucose metabolism. While exercise and dietary interventions have shown some transient benefits in reducing CMD risk, they often fail to improve clinically relevant disease markers and cardiovascular events. Moreover, SCI also places caregiving demands on their caregivers, who themselves experience health and functional decline. This underscores the need for more substantial interventions that incorporate appropriate physical activity, heart-healthy nutrition, and behavioral support tailored to the SCI population. Objectives: This randomized clinical trial (RCT) protocol will (1) assess the health and functional effects, user acceptance, and satisfaction of a 6-month comprehensive therapeutic lifestyle intervention (TLI) adapted from the National Diabetes Prevention Program (DPP) for individuals with chronic SCI and (2) examine the impact of a complementary caregiver program on the health and function of SCI caregivers and evaluate user acceptance and satisfaction. Caregivers (linked with their partners) will be randomized to ‘behavioral support’ or ‘control condition’. Methods: Dyadic couples comprise individuals with SCI (18–65 years, >1-year post-injury, ASIA Impairment Scale A-C, injury levels C5-L1) and non-disabled SCI caregivers (18–65 years). Both groups undergo lock-step circuit resistance training, a calorie-restricted Mediterranean-style diet, and 16 educational sessions focused on diet/exercise goals, self-monitoring, psychological and social challenges, cognitive behavioral therapy, and motivational interviewing. The outcome measures encompass the cardiometabolic risks, cardiorespiratory fitness, inflammatory stress, multidimensional function, pain, life quality, independence, self-efficacy, program acceptance, and life satisfaction for SCI participants. The caregiver outcomes include multidimensional function, pain, quality of life, independence, and perceived caregiver burden. Discussion/Conclusions: This study evaluates the effects and durability of a structured, multi-modal intervention on health and function. The results and intervention material will be disseminated to professionals and consumers for broader implementation. Trial Registration: ClinicalTrials.gov, ID: NCT02853149 Registered 2 August 2016.

## 1. Introduction

Chronic spinal cord injury (SCI) is associated with increased risk for cardiometabolic disease (CMD), all-cause cardiovascular disease (CVD), and type 2 diabetes mellitus (T2DM) [1,2]. Pervasive risk factors for these diseases are commonly observed after SCI and are associated with a sedentary lifestyle, hypercaloric nutrition relative to daily need [3,4,5,6], a stark loss of lean tissue [7,8,9,10,11], increased adiposity [12,13], and disrupted regulation of glucose and insulin [14,15]. The Consortium for Spinal Cord Medicine Clinical Practice Guideline for Cardiometabolic Disease Identification and Management identified obesity as the most prevalent cardiometabolic component risk [16], affecting 55–95.7% of the SCI population [17,18]. Alarmingly, obesity is known to pose a more substantial health hazard than the other CMD risks [19,20,21].

The current approach for the management of CMD risks after SCI primarily focuses on exercise rehabilitation and, to a lesser extent, nutritional modification. Studies of moderate-to-intense upper-extremity exercise (70–80% of peak capacity) [22,23,24] and circuit resistance training [25] report reduced individual CMD risks after a short-term intervention. However, the overall benefits typically fail to reach clinically relevant levels or test the sustainability of the intervention. A more reasoned view recognizes the need for a more comprehensive lifestyle intervention for the effective management of cardiometabolic risk factors in individuals with SCI. This requires a multidisciplinary approach incorporating population-appropriate physical activity, ‘heart-healthy’ nutrition at caloric levels that maintain stable body mass, behavioral support to sustain user engagement and long-term compliance, and close monitoring of cardiovascular health indicators [26].

Therapeutic lifestyle intervention (TLI)—involving a combination of physical activity, healthy nutrition, stress management techniques, and educational interventions—typically results in improved overall health and well-being. To address these needs, the Diabetes Prevention Program (DPP) was a landmark NIH-sponsored, 27-center randomized clinical trial that tested a lock-step lifestyle intervention program for persons who were at risk of developing T2DM. The DPP was found to be effective for promoting body mass reduction and lessening the conversion rates to frank diabetes [27,28] and was favorably compared against first-line pharmacotherapy for T2DM [29]. Importantly, programmatic modification of the DPP and testing of the long-term follow-up have shown remarkable success in sustaining—or even further improving—the benefits obtained during the initial treatment. The existing evidence similarly suggests an important role for ‘weight-lowering and fitness-promoting nutrition and exercise’ lifestyle interventions to improve the health and function of people aging with disability [30,31]. Previous studies have emphasized the need for weight management in SCI [32,33], and notably, a preliminary case series in which the DPP was refashioned for persons with SCI found that a clinically significant loss of body mass effectively reduced the component risks for CMD and diabetes [26]. In this way, TLI may be a promising approach for CMD management in SCI that merits further research to fully understand its potential benefits, sustainability, and optimal implementation strategies.

The health state of persons with SCI has recently been shown to impact their caregivers, as they both are living and aging with a disability. As current-day adjuvant providers, caregivers are challenged with assisting daily activities and promoting healthy living for individuals aging with SCI, while they may be similarly experiencing functional and health decline accompanying their own aging [34]. In general, the caregivers of individuals who are dependent on wheelchairs face greater physical demands, frequently needing to engage in activities such as lifting, pushing, or rolling an impaired body. Additionally, they often assume caregiving responsibilities at an earlier stage compared to caregivers of individuals with other physical disabilities. Unfortunately, healthcare plans and research efforts tend to primarily focus on the person with the disability, neglecting the inclusion of caregivers. As a result, it is not surprising that caregivers of individuals with spinal cord injuries (SCIs) experience high levels of pain, anxiety, and depression [35]. While there are limited lifestyle intervention programs targeting cardiometabolic health in SCI, there is an even greater scarcity of programs addressing caregiver health. To the best of our knowledge, there is early evidence supporting the benefits to the care-receiver when a caregiver participates in a linked behavioral intervention program [36]. Consequently, the concept of a coordinated intervention between the caregiver and care-receiver, where both partners can mutually benefit from the intervention, presents an intriguing therapeutic paradigm for consideration.

## 2. Objectives

I. Determine the health and functional impact, and user acceptance and satisfaction, of a 6-month comprehensive TLI adapted from the CDC National DPP in people with chronic SCI;
II. Determine the impact and user acceptance/satisfaction of a complementary caregiver curriculum (CCC) program on SCI caregiver health and function.
III. Determine whether the CCC enhances the health and functional benefits obtained by the SCI dyadic partner enrolled in the TLI program.

## 3. Hypothesis

I. Participants with SCI will have (a) lower body mass, (b) reduced cardiometabolic risks and inflammatory stress, (c) improved cardiorespiratory fitness and strength, (d) improved multidimensional function, pain, quality of life, and self-efficacy, (e) overall program adherence, and (f) greater life satisfaction following TLI;
II. Caregivers of persons with SCI will have (a) lower body mass, (b) reduced cardiometabolic risks and inflammatory stress, (c) improved cardiorespiratory fitness and strength, (d) improved multidimensional function, pain, quality of life, and independence, and (e) reduced perceived caregiver burden, following TLI;
III. Participants with SCI will have better outcomes on all of their tested hypotheses if their caregiver partner receives the CCC and not the control curriculum.

## 4. Materials and Methods

### 4.1. Trial Design

**Figure 1:**
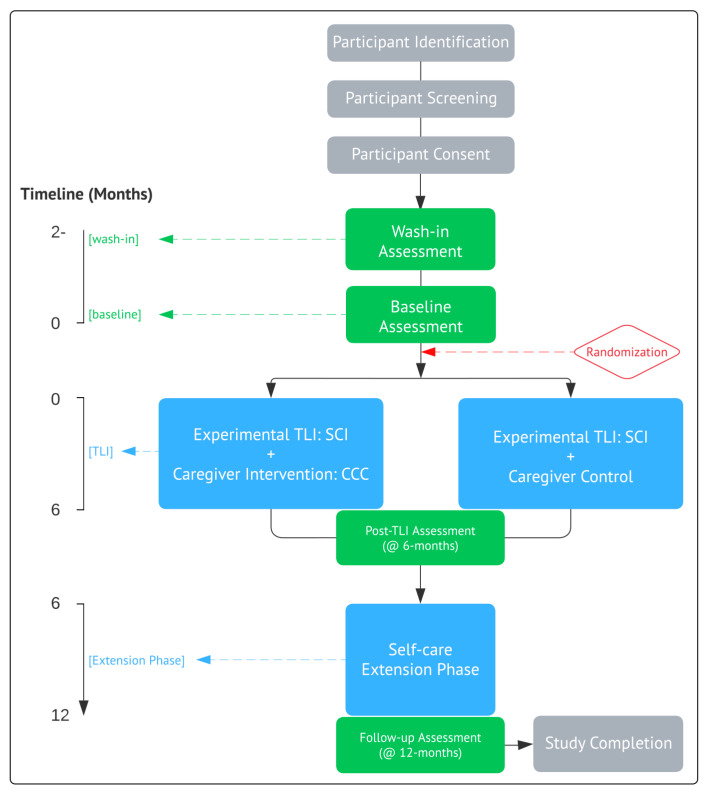
Trial Design.

This is a prospective randomized, controlled, multi-center intervention adopting a within-subjects repeated measures design, examining persons with disability from SCI and their caregivers who will be co-enrolled as dyadic partners. Following eligibility screening, consent, and randomization (of caregivers only), participants will attend two preliminary sessions including wash-in and baseline assessments (serving as ‘own-control’) prior to beginning in one of the two intervention conditions. Two post-intervention assessments will be conducted, one immediately post-TLI (6 months) and one follow-up (12 months). The trial design is summarized below (Figure 1), was approved by the Human Subjects Research Office, Miller School of Medicine, University of Miami (Institutional Review Board No. 20151065, dated 20 July 2016), and is registered as a controlled trial (ClinicalTrials.gov, ID: NCT02853149 on 2 August 2016).

### 4.2. Trial Setting

All human testing will take place at the lead center in the Lois Pope Life Center at the Miami Project to Cure Paralysis at the University of Miami’s Miller School of Medicine, and at a secondary specialty SCI rehabilitation facility at Craig Hospital (Denver, CO, USA). All biochemical analyses will take place at the Diabetes Research Institute, Division of Endocrinology, Diabetes and Metabolism at the University of Miami Miller School of Medicine.

### 4.3. Trial Recruitment

Candidates will be recruited by informal advertising, personnel referrals, and participant databases at both centers. The Miami Project maintains a database of individuals who have participated in past studies and volunteered additional participation. This is a searchable database under the management of the Director of Education. Institutional Review Board (IRB)-approved advertisements will be posted in the SCI/rehabilitation outpatient clinics at both centers. Basic information about the study status, such as active recruiting, closing, etc., will be searchable in the research section of each participating center’s website (http://www.themiamiproject.org/, (accessed on 2 August 2016); https://craighospital.org/programs/research/research-participation-opportunities, (accessed on 2 August 2016); and additional advertisements such as web blurbs and flyers will be distributed via emails and relevant social media accounts by each center. Upon initial contact, the study will be described and those interested in enrolling will be screened to ascertain eligibility. Written consent will be obtained during the initial on-site preliminary session, and participants will be informed that they can withdraw from the study at any time without consequence. Participants will be paid for their participation in this study. The total value of remuneration for participants at Miami will be USD 750, dispersed in four (4) payments: USD 150 after completion of screening and enrollment, USD 150 after 3 months post training, USD 150 after 6 months of training, and a balance of USD 300 after all data collection is completed at 12 months. The total value of remuneration for Craig participants will be USD 550, dispersed in four (4) payments: USD 100 after completion of screening and enrollment, USD 100 after 3 months post training, USD 100 after 6 months of training, and a balance of USD 250 after all data collection is completed at 12 months. This is primarily designed to reimburse travel expenses and thank them for any inconvenience caused. Compensation differences between sites reflect differences in the cost of living and compensation structures in place at each site to incentivize but not coerce participants.

### 4.4. Randomization

Given the elevated risk of CMD, the reported benefit of intervention studies on mitigating CMD risk [36], including a preliminary report on the TLI [26], and the ethical imperative to provide effective interventions [37], all participants with SCI will undergo a TLI program modeled after the CDC-supported widely adopted National DPP. The intervention program will incorporate population-targeted exercise, nutrition, and behavioral support in the form of a detailed curriculum, all aspects of which have been tested in a federal grant program at the lead center. Following the completion of baseline assessments, caregivers will be randomized to one of two groups using a stratified approach to achieve equal numbers in persons caring for enrolled partners with paraplegia and tetraplegia. Randomization will be performed independently within each stratum by an independent senior researcher using a list generated by the ‘RAND’ function in Microsoft Excel (Microsoft, Miami, FL, USA). Blinding is not achievable in this intervention study, and therefore neither participants nor research staff will be blinded. Participants are randomly sorted based on generated random numbers—a binary response variable, 0 for the control curriculum group and 1 for the CCC group— and their allocation to groups depends solely on their position in the sorted order. This randomization procedure ensures that participants are allocated to the CCC and control groups in an unbiased and unpredictable manner. Randomization of caregivers serves scientific and ethical purposes by allowing for rigorous assessment and ensuring equitable access to the intervention. Caregivers (linked with their partners) randomized to the test condition will undergo a CCC containing information that is similar to that presented to, but independently taught from, their partners. Caregivers randomized to the control condition will learn a time-matched (i.e., attention/time-controlled) curriculum containing generic information on the overall benefits of exercise and nutrition but lacking any specific exercise/nutritional guidelines, self-management strategies, or nutritional/meal composition. This information will be tailored to people without disabilities and include information the caregiver might otherwise find on the internet. All caregivers, irrespective of randomization, will receive SCI-specific material so that they can provide the appropriate support and best care possible in the framework of the TLI.

### 4.5. Trial Participants/Sample Size

The target sample size to be recruited is based on change in the primary outcome measure of body mass in the SCI group. Effect size was calculated based on statistical guidelines established by Cohen [38], similar SCI-related body mass reduction interventions [39], and ES in terms of relative risk reduction for the DPP [29]. Given a significant moderate ES of 0.48, for a two-tailed Fisher’s least significant difference test based on the difference between pre- and post-TLI body mass, with an α = 0.05 and power of 1-β ≥ 0.90, we calculated sample size (N) would require N ≥ 24 per group.

We targeted the enrollment of 30 dyadic couples (30 SCI; 30 caregivers = 60) who satisfy the inclusion and exclusion criteria (outlined below). We assumed an attrition of 20%, yielding an analytic sample size of 48. We note that preliminary data specific to this type of multi-modal and comprehensive TLI in SCI are not available for a direct calculation of ES. It is important to note that power analyses are estimates and assumptions about the effect sizes, and other parameters can vary. Additionally, other factors such as variability in participant characteristics, study design, and protocols can influence statistical power. In this study, we maintain alignment with standard statistical practice and fidelity to the DPP primary trial outcome, body mass, measured before and after the clinical intervention and extension phase.

#### Eligibility Criteria

**Inclusion Criteria:**

**Criteria**

**Description**
Male and Female with SCI/D > 1 year, aged 18–65 years.Individuals of both sex with spinal cord injury/dysfunction (SCI/D) for more than one year, aged between 18 and 70 years.ASIA Impairment scale (AIS) A-C Spinal Cord Levels C5-L1Individuals with AIS scale ranging from A to D and spinal cord levels C5-L1Anyone or more of the following:Participants must meet at least one of the following criteria:a. Waist circumference >94 cm (37 inches)Waist circumference exceeding 94 cm (37 inches).b. BMI ≥ 22 kg/m^2^ [40,41]Body Mass Index (BMI) equal to or greater than 22 kg/m^2^.c. Fasting dyslipidemia (HDL-C≤ 40 mg/dL (male)/≤50 mg/dL (female), OR; TG ≥ 150 mg/dL)Presence of fasting dyslipidemia, indicated by HDL-C ≤ 40 mg/dL (males) or ≤50 mg/dL (females), or TG ≥ 150 mg/dL.d. Linked with caregivers: male and female 18–70 years who consent to co-enroll in the study as partnersCaregivers aged between 18 and 70 years, of both sex, who are willing to participate alongside the study participant and provide social and/or physical support. Caregivers include family members, significant others, or friends living with a disabled (SCI) partner and offering personal assistance, routine emotional encouragement, and/or social interaction.


**Exclusion Criteria:**

For participants with SCI:
**Criteria****Description**Participating in at least 30 min of moderate-intensity physical activity for at least three months.Individuals engaged in moderate-intensity physical activity for a minimum of 30 min on at least three days per week for three months.Nutrition involving moderate caloric restriction for at least 6 months resulting in weight loss/gain ≥ 10% of total body massIndividuals who have undergone moderate caloric restriction resulting in a weight loss or gain of 10% of their total body mass over a period of at least 6 months.Surgery within 3 monthsIndividuals who have undergone surgery within the past three months.Grade 3–4 pressure ulcer within 3 monthsIndividuals who have had grade 3–4 pressure ulcers within the past three months.Limb pain that limits exerciseIndividuals experiencing limb pain that restricts their ability to exercise.Recurrent acute infection or illnessIndividuals with a history of recurrent acute infection or illness.PregnancyPregnant individuals.Previous myocardial infarction (MI) or cardiac surgeryIndividuals with a history of previous myocardial infarction (heart attack) or cardiac surgery.

For caregiver participants:
**Criteria****Description**Participating in at least 30 min of moderate-intensity physical activity for at least three months.Caregivers engaged in moderate-intensity physical activity for a minimum of 30 min on at least three days per week for three months.Nutrition involving moderate caloric restriction for at least 6 months resulting in weight loss/gain of 10% within the preceding 6 monthsCaregivers who have undergone moderate caloric restriction resulting in a weight loss or gain of 10% of their total body mass within the past six months.Surgery within 3 monthsCaregivers who have undergone surgery within the past three months.Limb pain that limits exerciseCaregivers experiencing limb pain that restricts their ability to exercise.PregnancyPregnant caregivers.Previous myocardial infarction (MI), cerebrovascular accident (CVA) or cardiac surgery that limits exerciseCaregivers with a history of previous myocardial infarction (heart attack), cerebrovascular accident (stroke), or cardiac surgery that limits their ability to exercise.Type I or II diabetes (by World Health Organization [WHO] criteria)Caregivers diagnosed with Type I or Type II diabetes according to World Health Organization (WHO) criteria.

Other exclusions applying to both groups:
**Criteria****Description**The following drugs: anti-hyperglycemic agents and lipid-altering agents within the past 6 monthsIndividuals using anti-hyperglycemic agents or lipid-altering agents within the past six months.Women who become pregnant will be advised to notify study personnelPregnant women will be instructed to inform study personnel of their pregnancy.Women must wait 6 months after child delivery to enter the studyWomen who have given birth must wait six months after delivery before entering the study.Individuals with a diagnosis of pre-diabetesIndividuals diagnosed with pre-diabetes according to American Diabetes Association (ADA) criteria.Traumatic brain injury (moderate/severe), substance abuse/dependence, major psychiatric condition, or participation in another trial where exercise/diet/behavior therapy would be confoundedIndividuals with traumatic brain injury (moderate/severe), substance abuse/dependence, major psychiatric condition, or participation in another trial that could confound exercise, diet, or behavior therapy.

We note that study material will be provided in English and Spanish, and personnel will be proficient in English and Spanish, to align with the representation of our study population.

## 5. Outcome Measures and Assessments

The trial outcome measures and assessments, timepoints, and participant time burdens are summarized in Table 1.

### 5.1. Description of Testing for SCI Participants

1. Anthropometry: Primary Outcome: Body Mass (BM): Participants will be weighed both in and out of their wheelchairs on a calibrated wheelchair scale, and the outcome of mass will be expressed as the average of the two measurements. Height/Waist Circumference/BMI: Height will be measured on a plinth to the nearest 0.25 inch. Waist circumference will be measured using a Gulick tension-regulated tape measure at the level of the umbilicus and recorded to the nearest 0.25 inch. Height [m] and weight [kg] will be used to calculate the body mass index (BMI) in kg/m^2^.
2. Cardiorespiratory Endurance: Cardiorespiratory endurance will be assessed as the peak oxygen consumption (VO_2_peak) on an arm crank ergometer using the open circuit spirometry method. Participants will refrain from exercise for 24 h prior to testing. Ten minutes of quiet rest will follow the preparation and precede the exercise to acquire the baseline values. A peak continuous graded exercise test will determine the VO_2_sub-peak/peak, HRsub-peak/peak, and ratings of perceived exertion (RPE; categorical ratio (0–10) scale) at subpeak and peak work. The participants will perform work using a calibrated electronically braked arm crank ergometer (Angio, Lode BV, Groningen, The Netherlands), or the equivalent heart rate and oxygen consumption will be recorded continuously from baseline through recovery. HR will be measured with standard electrocardiography, while expired respiratory gases will be collected and analyzed with an online open-circuit metabolic cart (Vmax ENCORE, CareFusion, San Diego, CA, USA) or equivalent. The participants will position their wheelchair at a distance from the arm crank ergometer where their arms can remain slightly flexed at the furthest point from the trunk, and wedges will be placed under the rear wheels to restrict chair movement. Individuals with tetraplegia will have their hands affixed to the arm cranks with hand wraps and athletic tape. Thereafter, the participants will begin cranking on the arm crank ergometer at an initial load of 5 W and will be instructed to maintain a cadence of 55–65 rpm. A digital display will provide real-time revolutions per minute (rpm) feedback.

In one-minute work stages [42], the resistance will increase at a rate of 5 W for all women and individuals with tetraplegia, or 10 W for individuals with paraplegia. The participants will continue until volitional fatigue, manifesting as either a verbal or nonverbal communication of the desire to stop or an inability to maintain cadence above 55 rpm. Upon cessation, participants will rest quietly for 10 min. The exercise termination will be based on the American College of Sports Medicine Guidelines for Exercise Testing and Training [ACSM, 9th Edition], with peak work defined as volitional exhaustion, the inability to maintain the targeted workload, or when increasing the workload fails to further increase VO_2_. The expired gases will be continuously analyzed using an open circuit indirect calorimetry system (Encore^®^, with Cardiosoft^®^ EKG monitoring, VIASYS, Inc., (Conshohocken, PA, USA) or equivalent). EKG rhythm will be continuously monitored to screen ischemia, rhythm disturbances, and signs/symptoms of exertional intolerance.

Perceived exertion (Borg Categorical-Ratio (0–10) Scale) will be obtained at the end of each exercise stage and at test termination.

3. Strength: Strength will be measured as upper-extremity dynamic strength on a Helms Equalizer 7000 multi-station exerciser (Helms Distributing, Polson, MT, or equivalent resistance equipment) using the same maneuvers adopted for training (Table 2). Subjects will be instructed to perform eight repetitions of each maneuver with each repetition lasting six seconds (3 s concentric, 3 s eccentric). If eight repetitions are completed in a controlled fashion, the weight will be increased and the exercise repeated. Incremental increases in weight (5 kg each for paraplegia, 2.5 kg for tetraplegia) will be added until eight controlled repetitions cannot be completed. The 1-RM will be calculated by the Mayhew regression equation [43], as we have previously reported [26]:
  1-RM = Wt/(0.533 + 0.419e(− 0.055 × Reps))
  where ‘1-RM’ is the calculated one-repetition maximum strength, “Wt” is the resistance used in the last set, in which more than three repetitions but less than eight repetitions were completed, and ‘reps’ equals the number of repetitions completed in the last set of testing. We note that these strength assessments are safe, relevant to daily activities, reliably measurable, and align with the goals of promoting independence and enhancing quality of life in individuals participating in the TLI.

4. Insulin Resistance, CVD Risk, and Inflammatory State: The participants will abstain from caffeine and alcohol consumption for 24 h before testing and will be tested in the post-absorptive state (8 h overnight fast and 48 h after the last exercise bout). Blood will be drawn into citrate and Gel and Lysis Activator (serum) tubes between 8:00– and 10:00 AM. The Biomarker and Immunoassay Laboratory at UM will be used to process the samples. We will use internet freeware (www.dtu.ox.ac.uk (accessed on 2 August 2016)/homacalculator/download.php) to calculate the second-generation Homeostatic Model Assessment 2 (HOMA2), a paradigm method used for assessing β-cell function and insulin resistance (IR) from basal (fasting) glucose and insulin or C-peptide concentrations. The HOMA2 model has been validated against physiological methods, compares favorably with other models (i.e., euglycemic clamp, minimal model, and ISI0-120), and requires only a single plasma sample assayed for insulin and glucose. The sample will also be assayed using automated methods for hemoglobin A1C. To assess the global CVD risk, we will use the proxy of the TC:HDL ratio (and, secondarily, the LDL:HDL ratio for dyslipidemia risk and the TG:HDL ratio for insulin resistance, respectively), which has predictive power for future CVD risk) that approaches the hazard forecasting of the Framingham regression equation. The assays for TC, TG, and HDL-C will be performed using automated methods and commercially available kits according to manufacturers’ instructions and run procedures [25]. HDL-C will be assayed after the precipitation of ApoB-containing lipoproteins. LDL-C will be computed using the method of Friedewald [44,45].

To test proatherogenic inflammatory stress, we will assess inflammatory biomarkers as surrogates for systemic inflammatory stress profiles as measured by cytokine and antibody production and gene expression in blood serum (inflammasome formation/activation and cytokine (TNF-α, IL-1β, and IL-6) production) using Western blotting and commercially available ELISAs.

5. Dietary Record: To track caloric intake and expenditure and ascertain the potential dietary drift in the caregiver control arm, participants will be instructed to complete and return a sample 4-day dietary record, including two representative weekdays and two weekend days to account for the changes in eating habits. As outlined by the DPP, physical food logs will be provided weekly, where habitual food and drink consumption are recorded, and the logs will be returned to the study personnel. The data will be analyzed at the lead center for total caloric intake and dietary composition, including discriminated macro/micronutrient content, using a nutritional software package (Food Processor II Windows v. 7.6; ESHA Research, Salem, OR, USA). The collected data will provide a comprehensive food intake assessment for scrutiny by both the investigators and study participants undergoing nutritional intervention.
6. (SCI) Function: We will operationalize ‘function’ as the participant-reported outcomes (PRO) of physical function. This refers to an individual’s capacity to carry out activities that require bodily movement. Most importantly, PROs capture their personal assessment of the function. PROs are defined as “a measurement of any aspect of a patient’s health status that comes directly from the patient,” and capture the impact of a disease or condition on the individual. We will administer the following:
  - SCI Functional Index (SCI-FI): Computer-adapted test (CAT short forms): The SCI-FI is a PRO that captures the activity limitations of persons with SCI [46,47]. It has six domains: basic mobility, self-care, fine motor function, ambulation, manual wheelchair, and power wheelchair. We will assess basic mobility and self-care in all participants and fine motor, ambulation, manual wheelchair, and power wheelchair as befits the individual’s injury and mobility characteristics. SCI-FI is patient-centered, accounting for the individual’s perspective on their own functioning, which is important for assessing the effectiveness of the behavioral component of the TLI. SCI-FI is widely recognized and accepted in both research and clinical settings. Its standardized assessment tools and scoring systems make it useful for comparing results across different studies and populations, enhancing the validity and reliability of research findings. Furthermore, SCI-FI is frequently used as an outcome measure in clinical trials and research studies focused on spinal cord injury and is validated for use in community-dwelling individuals with SCI [48].
  - Craig Handicap Assessment and Reporting Technique Short Form (CHART-SF): This measures the level of participation in a community setting. The CHART collects information on the degree to which the respondent fulfills the roles typically expected from people without disabilities. There are five dimensions that can be answered in quantifiable behavioral terms (e.g., hours of physical assistance, how much time is someone with you to assist you, how many relatives do you visit, etc.). For each CHART dimension, a scoring rubric allows a score from 0 to 100 points, the latter being the maximum attainable, which corresponds to a role fulfillment equivalent to that of most individuals without disabilities. We will focus our attention on the Physical Independence, Mobility, Occupation, and Social Integration Subscales. Extensive research has shown good levels of reliability and validity [49,50,51,52].

7. Pain: We consider that pain is multidimensional, and this is reflected in our choice of tests for assessment and classification. We will administer the following:
  - Multidimensional Pain Inventory-SCI Version (MPI-SCI): This is a psychometric instrument designed to assess pain and a range of psychosocial factors associated with chronic pain [53]. The answers are given on a numerical rating scale ranging from 0 to 6. The MPI-SCI consists of three sections: (1) pain impact; (2) perceived social support; and (3) activities. The internal consistency, stability, and validity of the MPI-SCI have been demonstrated in the SCI chronic pain population.
  - The International SCI Pain Basic Data Set (ISCIPBDS): This evaluates the worst, second worst, and third worst pain when a person experiences one or more pains [54]. The ISCIPBDS includes a pain classification made by a healthcare professional and self-reported information regarding the number of pains and self-reported information regarding the number of pain problems, location, intensity, and temporal pattern. It also assesses the pain interference with activities, mood, and sleep for each specific pain problem.
  - Neuropathic Pain Symptom Inventory (NPSI): This is sensitive to change and evaluates five common features of neuropathic pain [55]. The psychometric properties of the NPSI, including its sensitivity to change, provide a useful evaluation in clinical practice and clinical trials. The NPSI shows many similarities among different patient groups with peripheral or central lesions, which supports its utility as a method for pain evaluation in diverse neurotrauma populations.

The International Standards for Neurological Classification of Spinal Cord Injury will be administered before this assessment if one has not been performed within 2 years.

8. QoL, health perceptions, social functioning, and vitality. We will administer the following:
  - Spinal Cord Independence Measure-III (SCIM-III): This is a 19-item questionnaire assessing domains of 36 self-care, respiration and sphincter management, and mobility. The measure has been shown to have excellent internal consistency with α ranging from 0.77 to 0.85 [56]. The SCIM III correlates with Functional Independence Measure subscales with r values of 0.78 to 0.80 [57].

9. Treatment acceptance, life satisfaction, and self-efficacy. We will administer the following:
  - SCI Exercise Self-Efficacy Scale (ESES): A self-report to measure perceived exercise self-efficacy in individuals with SCI [58]. The scale requires individuals to indicate their confidence in performing physical activities and exercise. The scale is SCI-specific and measures perceived self-efficacy for various types of physical exercise. The measure has acceptable reliability and validity in SCI populations.
  - Credibility and Expectancy Questionnaire (CEQ): This is used to measure treatment expectancy. The CEQ demonstrates high internal consistency (*a* = 0.79–0.90). The retest reliability is *r* = 0.82 for the expectancy factor and *r* = 0.75 for the credibility factor. The items are rated based on cognitive appraisal and based on feelings about the therapy [59].
  - Satisfaction Questionnaire 9 (LSQ-9): This is used to assess whether the treatment improves life satisfaction. The nine-item version contains a single item assessing overall life satisfaction, along with eight additional items that are domain-specific. Normative data for chronic SCI have been obtained by Post et al. [60]. The measure has been used extensively in research with persons with SCI and has been shown to have excellent reliability and internal validity.

### 5.2. Description of Testing for Caregiver Participants

Prior to starting the training program, each subject will complete a basic fitness assessment. The data collected will be used to assess program progress and develop the initial exercise prescription. The testing procedures and order are as follows:

1. Anthropometry: Body mass will be measured to the nearest 0.1 kg using a digital platform scale. Height will be measured utilizing a wall-mounted measuring scale and recorded to the nearest 0.5 cm. Waist circumference will be measured using a Gulick tension-regulated tape measure at the level of the umbilicus and recorded to the nearest 0.5 cm. Height and weight will be used to calculate BMI as kg/m^2^.
2. Cardiovascular Fitness: *Bruce treadmill protocol* [61]: Subjects will wear a heart rate monitor to accurately determine their heart rate while exercising. After a 3 min warm-up at 1.7 mph/ 0% grade, the treadmill will be increased systematically as follows:

StageSpeed (mph)Incline (%)Duration (min)11.70321.710332.512343.414354.2163

3. Heart rate, Blood Pressure, and Rating of Perceived Exertion (RPE; categorical scale 6–20) will be measured 30 s prior to the end of each work stage. The test will be terminated when two consecutive stages elicit heart rates above 60% of the subject’s estimated maximal effort. The test will be terminated if the subject’s heart rate meets 85% of their estimated maximal heart rate. The estimated maximal heart will be calculated by the formula 208—(age × 0.66) (American College of Sports Medicine (ACSM), 2014) [62].
4. Cardiorespiratory Endurance is estimated by extrapolating the VO_2_ estimates achieved at the two consecutive submaximal steady-state heart rates to predict the maximum heart rate.
5. Strength: 1-RM strength for *chest press* and *leg press* will be estimated using the 7–10 repetition method and regression equation (see below). The resistance will be increased by 10 pounds when the high end of the repetition range can be completed before fatigue.

**Resistance Exercise**

**Prediction Equation for 7–10 RM Test**

**r**

**Adjusted R^2^**

**Chest Press**
−1.89 + (1.16 × Wt) + (1.68 × reps)0.950.91
**Leg Press/Extension**
95.00 + (0.65 × Wt) + (8.52 × reps)0.760.56

6. Insulin Resistance, CVD Risk, and Inflammatory State will be measured as described above for the SCI participants.
7. Dietary Record will be kept as described above for the SCI participants.
8. Function and Pain: We will administer the following:
  - Function: The four sub-domains of the PROMIS Physical Function CAT are *mobility* (lower extremity function), *dexterity* (upper extremity function), *axial* (neck and back function), and the *ability to carry out instrumental ADLs* [63].
  - West Haven–Yale Multidimensional Pain Inventory (MPI): This is a comprehensive instrument designed to assess a range of self-reported behavioral and psychosocial factors that are associated with chronic pain syndromes [64]. The MPI comprises three sections: Section 1 (Pain Impact), Section 2 (Responses by Significant Others), and Section 3 (Common Activities). The Pain Severity, Life Interference, Life Control, Affective Distress, Support, Negative Responses from Others, Solicitous Responses (SR) from Others, and Distracting Responses from Others subscales measure cognitive, affective, social, and behavioral responses. The remaining subscales assess the degree of participation in various types of daily activities: Household Activities Away from Home, Social Activities, and Outdoor Activities.

9. QoL, health perceptions, social functioning, and vitality. We will administer the following:
  - Credibility and Expectancy Questionnaire (CEQ): This is used to measure treatment expectancy. The CEQ demonstrates high internal consistency, (*a* = 0.79–0.90). The retest reliability is *r* = 0.82 for the expectancy factor and *r* = 0.75 for the credibility factor [59]. The items are rated based on a cognitive appraisal and based on the participants’ feelings about the therapy (e.g., how confident would you be in recommending this treatment to a friend who experiences similar problems?).
  - PROMIS—Satisfaction Social Satisfaction Short Form: The PROMIS Adult Satisfaction with Social Roles and Activities short form item bank assesses satisfaction with performing usual social roles and activities (e.g., “I am satisfied with my ability to participate in family activities”). We will be using the subset banks Satisfaction with Participation in Social Roles (v1.0) with revised item pools [65].

## 6. Intervention

### 6.1. SCI Participants

1. 6-Month Supervised Exercise Intervention: Circuit Resistance Training (CRT): The benefits of the CRT—a training algorithm adopted to increase muscular strength and cardiopulmonary endurance—have been described for persons with paraplegia [66,67] and tetraplegia [68]. Study participants will undergo CRT three times weekly on non-consecutive days or two consecutive days and one non-consecutive day for 24 weeks. An exercise technician will assist with the station setup during training. Each session will last approximately 40–45 min and employ resistance training (weightlifting) and endurance activities (reciprocal arm exercise, VitaGlide^®^ (VitaGlide, LLC., Rockledge, FL, USA), RehabMed International, or arm crank ergometry, Colorado Cycle) with interposed periods of incomplete recovery (i.e., heart rate not falling to baseline). The training session will follow a structured sequence. Prior to each session, a 2 min warm-up will be conducted on a Vita-Glide^®^ or arm crank ergometer. The resistance exercises will be performed in pairs, with each pair consisting of two maneuvers performed consecutively. Each maneuver will involve 10 repetitions, with a 6 s movement duration, divided into 3 s for concentric (lifting) and 3 s for eccentric (lowering) phases. Following the resistance exercises, there will be a 2 min period of endurance exercise without applied resistance. This pattern of alternating between resistance and endurance exercises will continue until the subjects complete three rotations through each resistance station. During the first two weeks of each month, the resistive loads for training will be set at 50% of the calculated 1-repetition maximum (1-RM) values obtained from the initial isoinertial strength testing, as previously reported [69]. Subsequently, the loads will be increased to 55% and 60% of the 1-RM during weeks three and four of each month, respectively. To account for the effects of training, the 1-RM for each maneuver will be recalculated during the final training session of every 4-week cycle. Please refer to Table 2 for a summary of the resistance maneuvers.

Modifications of the CRT for Tetraplegia: Given that the previously described protocol was originally developed and tested for individuals with full upper-extremity motor function, we have made adaptations to accommodate individuals with tetraplegia up to the C5 level [68]. These adaptations involve modifying the order of resistance maneuvers to reduce the time required for changing between the resistance stations. Additionally, we have adjusted the sequencing of resistance and endurance exercises, allowing them to be performed consecutively in contiguous time blocks. In our previous protocols for individuals with paraplegia, we alternated between resistance and endurance activities without providing rest intervals. However, to minimize the setup and transfer time for individuals with tetraplegia, we now instruct participants to complete all resistance exercises followed by all endurance activities within uninterrupted time blocks. The order of the exercises (i.e., resistance and endurance) will be alternated on each training day. Furthermore, to facilitate the station setup and changes, we will have an exercise technician available to assist the participants. These modifications ensure that individuals with tetraplegia can effectively engage in the adapted workout, optimizing their training experience.

For the endurance exercise component, we will utilize the Vita-glide^®^ arm ergometer (or a similar alternative) at a cadence of 50 revolutions per minute (rpm). The duration of each session will initially be set at 10 min and gradually increase to 20 min of continuous activity. To monitor the approximate work intensity, a Polar^®^ monitor will be employed, utilizing a target of 60% for the heart rate reserve (HRR). The Karvonen equation [70] will be utilized to calculate the target heart rate, taking into account both the resting and peak heart rates obtained from the arm-crank exercise tests. These values will be reassessed during the midterm evaluation to account for any changes resulting from the training effects. The use of the 60% intensity range (or the corresponding power output) for conditioning individuals with tetraplegia has been shown to significantly increase VO_2_peak after 8–10 weeks of arm conditioning [67]. By adjusting the work intensity based on the Karvonen equation, we can ensure the use of appropriate levels of cardiovascular conditioning throughout the training program. In those subjects without a normal heart rate response to exercise (due to sympathetic decentralization) [71], the Borg Rating of Perceived Exertion (RPE) scale [72] will be used as an ancillary measure to monitor work intensity. Sphygmomanometers will be ready and available in the circumstance of abnormal responses during exercise (i.e., autonomic dysreflexia) [73].

2. 6-Month Nutritional Intervention: The nutritional intervention for the trial will consist of a 24-week energy-restricted Mediterranean-style diet. In accordance with the DPP study [74] and ADA guidelines [75], we will ensure that the participants have the daily energy consumption necessary to maintain baseline body weight and consider physical activity energy expenditure. The daily energy intake will be reduced to achieve a 500–1000 kcal/d deficit. This is sufficient to result in a weight loss of 1–2 lbs/week and approximately 7% of baseline body mass by the end of the 24-week intervention. The approximate target daily energy intakes prior to the adjustment for physical activity will be 1200 kcal/d for subjects weighing 120–170 lbs at baseline, 1500 kcal/d for subjects weighing 175–215 lbs at baseline, 1800 kcal/d for subjects weighing 220–245 lbs at baseline, and 2000 kcal/d for subjects weighing more than 250 lbs at baseline. The experimental nutrition will emphasize fruits, vegetables, whole grains, and olive oil, while animal sources of protein will be restricted to poultry and fish. The daily energy intake will be composed of 45–50% carbohydrate, 15% protein, and 35–40% fat. The daily fat intake will consist primarily of monounsaturated fats (18–20% of the daily energy intake) from olive oil (30–50 g/d, ~2.2–3.7 tbsp) and tree nuts (<20 g/d), with only a small portion from saturated fats (<7%) and the balance from polyunsaturated fats (10–13%). This macronutrient composition closely resembles those used by several recent investigations of Mediterranean-style nutrition [76,77,78]. While the relative carbohydrate consumption appears low (45–50% of daily energy intake), a lower-carbohydrate Mediterranean-style diet (<50%) results in greater body weight losses among obese women than higher-carbohydrate Mediterranean-style diets (>50%). In accordance with ADA guidelines, the intake of trans-fat will be minimized, the daily cholesterol intake will be limited to <200 mg/d, and the fiber intake will be targeted at 14 g/1000 kcal consumed.

A nutritional education and assessment will teach subjects food intake self-monitoring skills by using scales, measuring cups, and spoons provided to them for home use. Nutrition booklets will be provided, which will include information on common food items, food groups, servings, sample day-to-day menus, recipes, and food logs.

3. 6-Month Behavioral Intervention: general principles: The core DPP intervention will involve a generalized 16-session protocol aimed at altering behavior through education, problem-solving skills training, and cognitive restructuring (Table 3). The intervention will be delivered by a lifestyle coach assigned to the participant, primarily in a one-on-one format that allows tailoring of the intervention to the needs of the individual. To accommodate the specific demands of an SCI population, the general framework of the DPP training protocol will be replicated with modifications tailored to an SCI population. The complete curriculum is summarized in Table 3. The intervention structure consists of a total of 16 training sessions that cover various aspects such as education, nutrition, exercise, goal setting, and the self-monitoring of food intake and physical activity. The first eight sessions focus on providing information and strategies to address the psychological, social, and behavioral challenges associated with maintaining a behavior change. The remaining eight sessions concentrate on education, nutrition, exercise, outlining goals, and self-monitoring. Each training session will have an approximate duration of 30–60 min. To support the participants’ progress, each individual will receive a personalized lifestyle intervention manual. This manual will include their screening and pre-intervention assessment data, goals, key training elements, and motivational messages tailored to their specific needs. The manual serves as a valuable resource throughout the intervention, providing guidance and support to participants as they work towards their lifestyle change goals.

4. 6-Month Self-care Extension-Phase Maintenance Program: The final (6th) month of the clinical training phase will be used to train participants in the extension phase of the intervention to facilitate the adherence and long-term success of the program. A thorough review of the data and outcomes, health metrics, compliance, and challenges will be assessed. The modifications and adjustments to exercise routines, dietary plans, and behavioral support strategies will be discussed. We have already published a Thera-Band^TM^ adaptation of the CRT program, which can be set up in the home and adapted for persons with tetraplegia. The use of this method was featured in CDC NCPAD News, July 2007 (*This Poor Man’s Exercise System*, Alan Troop). The lifestyle coaches will work with participants to train for the Thera-Band program and will set exercise-intensity thresholds that simulate those undertaken in the clinical phase of the training. Heart rate, RPE, and the talk test will be discussed as tools to monitor exercise intensity. The participants will be prepared to continue their exercise routines as prescribed.

### 6.2. CCC Participants

1. 6-Month Supervised Exercise Intervention: The following exercise training protocols are within the accepted guidelines established by the American College of Sports Medicine [62]. The caregiver intervention group will have a 6-month supervised exercise program at the Medical Wellness Center three times weekly that will last 40–45 min. The activities will include resistance training (weightlifting) and endurance training (treadmill and bicycles). The study personnel at the center will oversee the training and testing of the caregivers and establish the exercise prescription. The participants will work with a coach who will direct the exercise performed and work intensity. At the end of each month, the investigators will retest the strength and adjust the weight lifted to match the change in strength due to the training effects. The resistance training protocol consists of seven exercises (chest press, seated row, shoulder press, pulldown, bicep curl, seated dip, and leg press), performed on selectorized resistance training equipment. During the first six weeks, the subjects will be asked to complete one set of 12–15 repetitions of each exercise. When 15 repetitions can be completed comfortably, without fatigue, and in good form, the resistance will be increased by 5–10%. This progression will continue each time the upper end of the repetition range is successfully completed. After six weeks, the repetition range will be adjusted to 8–12 repetitions per set and finally increased to two sets of 8–12 repetitions for weeks 13–72. The cardiovascular exercise prescription will range from 50 to 70% of the estimated VO_2_max for 20–40 min. VO_2_max will be estimated from the data collected during the submaximal treadmill protocol completed during the baseline fitness assessment (i.e., Bruce protocol). Initially, the subjects will start at 50% of the estimated VO_2_max for 20 min and systematically progress to 70% of the estimated VO_2_max for 40 min over the course of the three months. They will then maintain the latter intensity and duration for the remainder of their exercise training program.
2. The 6-Month Nutritional Intervention will be carried out as described above for the SCI participants.
3. The 6-Month Behavioral Intervention will be carried out as described above for the SCI participants.
4. The 6-Month Self-care Extension-Phase Maintenance Program will be as described above for the SCI participants.

The Caregiver control group will follow a similar schedule but a different curriculum. Although they will have access to the same facilities (Medical Wellness Center) as the intervention group, the control caregiver participants will receive generic (“unstructured’) information about healthy eating and exercising without specific education on food types, exercise guidelines, caloric expenditure, or nutritional composition. The content sources will include online websites, such as *Web*MD Living Healthy (www.webmd.com/living-healthy, (accessed on 2 August 2016)), which typically provide generic information describing the need to exercise (but not providing an exercise prescription), eat well (but not providing a defined diet), and broad lifestyle recommendations. The study personnel at the Medical Wellness Center will oversee the testing of the caregivers without disability but will not establish any specific exercise prescription or training at any time of the intervention. The caregivers will be instructed by the Medical Wellness Center regarding the use of the facilities and safety guidelines.

## 7. Trial Safety and Adverse Events

Each trial site will have an assigned medical director to serve as the trial physician and oversee all aspects of participant medical care. The trial will also have an assigned independent medical monitor providing trial medical oversight and project evaluation plans. All testing will be performed at a medical center that is located near an emergency room, and all facilities and trial personnel are trained on the standing policies and procedures for emergencies. This will be designed to reduce the risks and burdens, with the further aim of reducing the potential risks and burdens through strict adherence to best practices [79].

Any adverse events or serious adverse events that occur during the study will be promptly reported to the Institutional Review Board (IRB) within the required timeframe. In the event of an adverse event, the principal investigator will immediately inform and seek advice from the study physician. The study physician will assess whether the event(s) can be attributed to the study procedures outlined in the protocol. The evaluation of adverse events by the study physician will be conducted using the following criteria [80]:

i. Grade 1 (mild): Individuals experience an awareness of symptoms, which can be easily tolerated. These symptoms are typically transient and do not necessitate any specific treatment. They do not interfere with the individual’s usual or normal daily activities.
ii. Grade 2 (moderate): Symptoms may be improved with simple therapeutic measures. While they may interfere with the individual’s normal daily activities, they do not prevent them from participating in these activities.
iii. Grade 3: This level of adverse event represents an incapacitating event where the individual experiences an inability to perform their usual activities.
iv. Grade 4 (life-threatening/disabling): In this category, the participant is in a critical condition where there is a risk of death, worsening disability, or impairment compared to their pre-existing condition at the time of the event.

For adverse events categorized as grades 1 and 2, the investigators will closely monitor the participant and provide standard medical or therapeutic care as needed. If there are repeated instances of grade 1 and 2 events, the investigators may deem it necessary to notify the Institutional Review Board (IRB) and consider halting the study. In such cases, the investigators will take the appropriate action and inform the IRB accordingly. For grade 3 and 4 events, each occurrence will be individually assessed. If any grade 3 or 4 event takes place, it may prompt the investigators to notify the IRB and consider stopping the study. The investigators will follow the necessary steps and inform the IRB accordingly. In the absence of such circumstances, if the IRB determines that the serious adverse event is related to the protocol, the study will be halted and undergo evaluation to determine if it should continue.

## 8. Data Management

### 8.1. Electronic Data Records

All electronic files will be stored on password-protected computer terminals located in the Christine E. Lynn Rehabilitation Center for the Miami Project to Cure Paralysis, the University of Miami Miller School of Medicine, and Craig Hospital. Computer security is provided through data encryption, firewall protection, and data backup on the Miami Project server, accompanied by a DUO Multifactor Authorization. All source data obtained will be entered into a de-identified data bank and stored securely on the network.

### 8.2. Physical Data Records

Data will be stored in a locked filing cabinet, within a locked room, and behind two levels of access security at the Christine E. Lynn Rehabilitation Center and Craig Hospital. The source data from the laboratory and testing equipment are kept under the same level of access security described above.

### 8.3. Confidentiality

The identified data (name, date of birth, contact details, next of kin, and any protected health information (PHI) obtained) will be kept by the University of Miami and Craig Hospital. This data will be stored in a password-protected file and on a computer under the same level of access security described above. The deidentified study data, coded with a unique study ID assigned to each participant, will be kept by the University of Miami and Craig Hospital. The electronic records will be kept indefinitely, and the physical records will be kept for a minimum of 3 years after enrollment is closed and destroyed by the shredding services provided by each center. The participants’ samples will be stored in accordance with the Human Tissue Act 2004.

## 9. Dissemination

The data obtained from this trial will be submitted for peer-review publication and presented at professional conferences and meetings (American Spinal Injury Association and International Spinal Cord Society). Following any publication, we will deposit the final dataset in a data repository, ensuring that the final dataset is anonymized. Access to the data and procedures to release the data will be defined by the policies of the repository in which the data are placed.

## 10. Discussion

This study is designed to test the effects and durability of a structured multi-modal intervention on enhancing the health and function of persons with SCI and their caregivers. The expected results from this trial may serve as an important guide for long-term rehabilitation strategies for the cardiometabolic risk factors observed in patients with chronic SCI and the general health and quality of life of their caregivers. The recently published clinical practice guidelines on “Identification and Management of Cardiometabolic Risk after Spinal Cord Injury” [36] recommend lifestyle intervention as the primary management approach to CMD risk components after SCI. Moreover, the CMD prevention guidelines established by the American College of Cardiology/American Heart Association (ACC/AHA) state that one of the principal components of an “effective high intensity…lifestyle intervention” is the “use of behavioral strategies to facilitate adherence”, and it is asserted that this therapy should provide a “structured behavioral change program”.

Past reviews on CMD risks in SCI suggest the use of clinical pathways incorporating a comprehensive TLI to foster a more effective health-centered culture for those with SCI and their health care providers [3], and preliminary studies suggest that the TLI described in this trial can effectively reduce the component risks for CMD in SCI [26]. The results of this trial will have more widespread implications for the management and prevention of CMD in SCI and an effective cross-over of benefits for both the caregiver and care-receiver. Overall, this trial will enhance the validity and applicability of the current recommended guidelines for CMD risks in SCI and the growing health concerns for their caregivers.

## 11. Conclusions

This study is designed to systematically test the effects and durability of a structured multi-modal intervention on enhancing the health and function of people with SCI and their caregivers. The RCT results will be presented to and published for professionals and consumers, and the detailed intervention training and materials will be made available for widespread dissemination.

## Figures and Tables

**Table 1:** Overview of outcome measures and assessment, timepoints, and participant time burdens for testing.

Variable	Measure/Data Source	Study Month						Time (min)
		−2	0	2	4	6	12	
SCI Testing								
Body Mass	Calibrated Scale	◾️	◾️	◾️	◾️	◾️	◾️	5
Fitness: Endurance	Peak Oxygen Uptake	◾️	◾️			◾️	◾️	40
Fitness: Strength	Composite Strength: Mayhew Prediction Model	◾️	◾️			◾️	◾️	30
Insulin Resistance	HOMA2	◾️	◾️			◾️	◾️	10
Global CVD Risk	TC:HDL Ratio	◾️	◾️			◾️	◾️	
Inflammatory State	TNF, IL1, IL6	◾️	◾️			◾️	◾️	
Dietary Record	Food Log (4 consecutive days)	◾️	◾️			◾️	◾️	20
SCI Function	SCI Functional Index (SCI-FI)—6 Domains	◾️	◾️			◾️	◾️	<10
Participation	Craig Handicap Assessment and Reporting Technique (CHART)	◾️	◾️			◾️	◾️	15
Basic Pain and Classification	The International SCI Basic Pain Data Set (ISCIBPDS)	◾️	◾️	◾️	◾️	◾️	◾️	10
Neuropathic Pain	The Neuropathic Pain Symptom Inventory (NPSI)	◾️	◾️			◾️	◾️	5
Multidimensional Pain	The West Haven–Yale Multidimensional Pain Inventory (MPI-SCI)	◾️	◾️			◾️	◾️	15
Independence	Spinal Cord Independence Measure-II (SCIM-II)	◾️	◾️			◾️	◾️	20
Self-Efficacy	SCI Exercise Self-Efficacy Scale (SCI_ESES)	◾️	◾️			◾️	◾️	5
Treatment Acceptance	Credibility and Expectancy Questionnaire (CEQ)	◾️	◾️			◾️	◾️	5
Life Satisfaction	Life Satisfaction Questionnaire-9 (LSQ-9)	◾️	◾️			◾️	◾️	5
Caregiver Testing								
Body Mass	Calibrated Scale	◾️	◾️	◾️	◾️	◾️	◾️	5
Fitness: Endurance	Prediction Equation	◾️	◾️			◾️	◾️	40
Fitness: Strength	Composite Strength: Mayhew Prediction Model	◾️	◾️			◾️	◾️	30
Insulin Resistance	HOMA2	◾️	◾️			◾️	◾️	10
Global CVD Risk	TC:HDL Ratio	◾️	◾️			◾️	◾️	
Inflammatory State	TNF, IL1, IL6	◾️	◾️			◾️	◾️	
Dietary Record	Food Log (2 weekdays + 2 weekend days)	◾️	◾️			◾️	◾️	20
Caregiver Function	PROMIS—Physical Function—All Domains	◾️	◾️			◾️	◾️	<10
Multidimensional Pain	The West Haven–Yale Multidimensional Pain Inventory (MPI-SCI)	◾️	◾️			◾️	◾️	15
Life Satisfaction	PROMIS—Social Satisfaction Roles Domain	◾️	◾️			◾️	◾️	5
Anxiety and Depression	Hospital Anxiety and Depression Scale (HADS-A) Anxiety Subscale	◾️	◾️			◾️	◾️	20
Treatment Acceptance	Credibility and Expectancy Questionnaire (CEQ)	◾️	◾️			◾️	◾️	5

**Table 2:** Description of resistance maneuvers used in the CRT intervention.

Exercise Maneuver	Description
Military Press	Shoulder abduction with scapular elevation and upward rotation starting from the fully adducted and depressed position.
Horizontal Row	Shoulder horizontal abduction with scapular adduction starting from a position of maximum forward reach.
Pec Dec	Shoulder horizontal adduction while in external rotation to the midline from the maximum tolerated horizontal abduction in external rotation.
Preacher Curl	Elbow flexion supported on an inclined pad from the fully extended position.
Latissimus Pulldowns	Shoulder adduction with scapular downward rotation and depression starting from the maximal upward reach position.
Seated Dips (“Rickshaw”)	Shoulder flexion, scapular depression, and elbow extension while maintaining arms as near the body as possible, from the fullest allowed point of shoulder joint extension, scapular elevation, and elbow flexion.

Abbreviations: CRT; circuit resistance training.

**Table 3:** Curriculum summary for behavioral intervention.

	Core Intervention Training Curriculum.	
	Session	Topic
Focus is on diet and exercise goals and education	1	Introduce lifestyle intervention. Explain study goals.
	2	Introduce self-monitoring of weight at home.
	3	Teach **3** ways to eat less fat.
	4	Educate about healthy eating. Recommend alternate foods.
	5	Introduce physical activity modules.
	6	Tailor physical activity regimen to needs of the individual.
	7	Teach principles of energy balance between calories and exercise.
		Teach principles of health maintenance from exercise.
	8	Introduce principles of stimulus control as a method to prevent unhealthy eating.
		Introduce principles of stimulus control as a method to maintain exercise adherence.
Focus is on psychosocial and behavioral strategies	9	Present five-step model of problem solving.
	10	Introduce basic skills for eating and exercising away from home.
		Introduce basic skills for exercising away from home.
	11	Practice identifying negative thoughts and how to counter them.
	12	Introduce concept that slips are part of lifestyle change and provide tips for behavioral-change maintenance.
	13	Introduce principles of aerobic fitness and coping with boredom.
	14	Provide strategies for managing social cues, both stressful and supportive.
	15	Summarize stress management principles presented over the course of the intervention.
	16	Focus on enhancing motivation and maintaining behavioral change post-lifestyle intervention.
